# MicroRNAs are inappropriate for characterising hearing impairment in mitochondrial disorders

**DOI:** 10.1186/s13023-018-0831-5

**Published:** 2018-05-31

**Authors:** Josef Finsterer, Sinda Zarrouk-Mahjoub

**Affiliations:** 1 Krankenanstalt Rudolfstiftung, Postfach 20, 1180 Vienna, Austria; 2 Pasteur Institute of Tunis, University of Tunis El Manar and Genomics Platform, Tunis, Tunisia

**Keywords:** Mitochondrial, Hereditary neuropathy, Phenotype, Genotype, Multisystem disease, Lactic acidosis

## Letter to the Editor

We read with interest the review by Di Stadio et al. about microRNAs (miRs) for the diagnostic work-up of hypoacusis in MELAS [1]. It was suspected that miR-34a, miR-29b, miR-299-3p, and miR-431 can be useful as biomarkers for detecting and characterising inner ear involvement in MELAS [1]. We have the following comments and concerns.

We do not agree with the statement that “MELAS shows the highest incidence of hearing loss” among mitochondrial disorders [1]. There is another specific mitochondrial syndrome in which per definition every patient presents with hearing impairment, known as maternally inherited diabetes and deafness (MIDD) [2]. Additionally, hypoacusis is frequently found coenzyme-Q deficiency [3, 4].

We also do not agree with the statement that “cochlear conduction cannot be responsible for hearing impairment in MELAS” [1]. Mitochondria are present in nearly all cell types in high amounts, except for erythrocytes. Mitochondrial disorders (MIDs), including MELAS, may even manifest in the bone marrow, presenting as Pearson syndrome or other types of anemia [5] and, additionally, in sensory cells of the cochlea and mucosal cells of which the middle ear is fully lined with.

Hearing impairment in MIDs may not only be due to a peripheral perception or conduction problem but also due to a central conduction or processing defect. The organ most frequently affected in MELAS is the brain (epilepsy, stroke-like episodes, basal ganglia calcification, ataxia, atrophy, confusion, dementia, psychosis, leukoencephalopathy), this is why central causes of hypoacusis should be always considered. Were central causes of hearing impairment excluded in all studies cited in the review article?

miRs are currently pushed and endeavoured as biomarkers or as etiologic or pathogenic factors of various different disorders. However, miR levels are not only abnormal in genetic and inflammatory diseases or ageing but also in malignancies (e.g. prostate cancer), metabolic conditions (e.g. diabetes), or neurodegenerative disorders (e.g. ALS) [6]. For example, miR-29b has been involved in the etiology of prostate cancer [7], miR-34a promotes cell cycle arrest and apoptosis and suppresses cell adhesion in osteosarcoma [8], miR-299-3p has been shown to promote cell growth and to regulate G1/S transition in promyelocytic leukemia [9], and miR-431 is known to regulate axon regeneration in mature sensory neurons [10].

Additionally, miR levels may not only be influenced by various pathologic conditions but also affected by a number of drugs, such as statins [11]. Thus, we should be informed about the current medication of patients discussed in Dr. Di Stadio’s review. It is also essential that we know how many of the discussed patients had diabetes, a frequent manifestation of MIDs, including MELAS.

The authors also do not address the effect of heteroplasmy on miR levels and on the variable phenotypes of temporal bones and the cochlea. Also the influence of ageing on the miRs levels was not discussed. We should be informed about the age range of the included patients and if miR levels were dependent on age.

Overall, it remains speculative if miRs influence the phenotype of patients carrying mutations in the mtDNA or whether they reflect disease activity or severity, including hearing function in MELAS. Due to their multifactorial involvement, there is currently no evidence for the usefulness of miRs as reliable biomarkers for disease activity, severity, and progression, or whether they have a pathogenic effect in MELAS [12].

## Abbreviations

MELAS: Mitochondrial encephalopathy lactic acidosis and stroke-like episodes
MID: Mitochondrial disorder
MIDD: Maternally inherited diabetes and deafness
